# CT Guided Needle Biopsy of Peripheral Lesions–Lesion Characteristics That May Increase the Diagnostic Yield and Reduce the Complication Rate

**DOI:** 10.3390/jcm10092031

**Published:** 2021-05-09

**Authors:** Manabu Tajima, Shinsaku Togo, Ryo Ko, Yoshika Koinuma, Issei Sumiyoshi, Masahiro Torasawa, Nao Kikuchi, Akihiko Shiraishi, Shinichi Sasaki, Shinsuke Kyogoku, Ryohei Kuwatsuru, Kazuhisa Takahashi

**Affiliations:** 1 Division of Respiratory Medicine, Juntendo University Faculty of Medicine & Graduate School of Medicine, 3-1-3, Hongo, Bunkyo-ku, Tokyo 113-8431, Japan; mntajima@juntendo.ac.jp (M.T.); rkou@juntendo.ac.jp (R.K.); ymatsuda@juntendo.ac.jp (Y.K.); i-sumi@juntendo.ac.jp (I.S.); kztakaha@juntendo.ac.jp (K.T.); 2 Department of Respiratory Medicine, Juntendo University Urayasu Hospital, 2-1-1, Tomioka, Urayasu, Chiba 273-0021, Japan; m-torasawa@juntendo.ac.jp (M.T.); ssasaki@juntendo-urayasu.jp (S.S.); 3 Department of Radiology, Juntendo University Urayasu Hospital, 2-1-1, Tomioka, Urayasu, Chiba 273-0021, Japan; nakikuti@juntendo.ac.jp (N.K.); skyogoku@juntendo.ac.jp (S.K.); 4 Department of Radiology, Juntendo University Faculty of Medicine, 2-1-1, Hongo, Bunkyo-ku, Tokyo 113-8421, Japan; asiraisi@juntendo.ac.jp (A.S.); kuwaturu@juntendo.ac.jp (R.K.)

**Keywords:** bronchoscopy, CT-guided needle biopsy, lung cancer, metastatic lung tumor, thoracic malignancy, transbronchial biopsy

## Abstract

Computed tomography-guided needle biopsy (CT-GNB) has a high diagnostic yield for lung cancer but higher complication rates compared to those of other biopsy modalities. We sought to clarify in which thoracic lesions we could achieve a quick pathological diagnosis using CT-GNB, considering the risks and benefits. We retrospectively enrolled 110 patients who underwent CT-GNB and 547 patients who underwent transbronchial biopsy (TBB) for parenchymal lung lesions in clinical practice. The diagnostic rates of CT-GNB and TBB were 87.3% and 75.3%. After failed diagnosis with other biopsy modalities, 92.3% of patients were finally diagnosed using CT-GNB and 65.8% using TBB. In cases with a negative bronchial sign, there was a statistically higher diagnostic rate with CT-GNB than with TBB (*p* < 0.001: 89.4% vs. 0%). Complication rates were higher with CT-GNB (50.9%) than with TBB (16.3%). However, there were lower rates of complications in cases with inhomogeneous tumors, subpleural lesions, and when more than 15 mm of the punctured needle length was within the target. We conclude that CT-GNB is an effective biopsy modality with a high diagnostic rate that is especially recommended when the bronchus sign is negative. It can be safely performed if risk factors for complications are taken into account.

## 1. Introduction

Biopsies are essential for identifying and confirming a definitive pathological diagnosis of lung cancer. Reliable biopsy approaches that can be used to make accurate early diagnoses are particularly important, as this can improve long-term survival. Conventional flexible bronchoscopy is a safe diagnostic modality compared to computed tomography-guided needle biopsy (CT-GNB) [1]. However, it has a low diagnostic yield that depends on various factors including the size, location, and appearance of the target on imaging, which can be particularly problematic in cases such as peripheral parenchymal lung lesions [2,3,4]. In addition, some radiological features of the target lesion, such as ground glass opacity (GGO), can be difficult to detect through radiography as the biopsy target [5,6]. Tumors that contain necrotic tissue within and reveal inhomogeneous density in the CT image may affect to select the diagnostic yield with bronchoscopy. In clinical practice, the bronchus sign is the presence of a bronchus inside the target. When this is identified on pre-biopsy CT images, it is important to choose bronchoscopy as the first diagnostic modality because of the higher diagnostic rate [7]. However, when it is unclear whether the bronchial sign is positive, the pulmonologist sometimes compromises on the choice of bronchoscopy because of its low complication rate.

CT-GNB is a well-established, reliable procedure with a high diagnostic yield (77–96%) [8,9,10,11,12], but it requires radiation exposure and has a high complication rate [13,14,15]. The major complications with CT-GNB are post-procedural pneumothorax, hemorrhage, and hemoptysis [16,17]. Severe complications may require further treatment, such as drainage of a pneumothorax, and intubation or blood transfusion with hemoptysis. However, few studies have addressed when CT-GNB should be selected according to the radiological features of the target lesion, while considering the risks and benefits in comparison to those of other diagnostic modalities. Moreover, it has not yet been established whether CT-GNB should be the preferred modality for patients in whom other modalities failed to provide a diagnosis. There are no strict criteria to guide the choice between CT-GNB and transbronchial biopsy by bronchoscopy (TBB), and no established guidelines for general clinical practice. In this study, we retrospectively investigated cases from our institution to identify trends in our clinical practice and we looked for lung nodule characteristics (size, specific structure and location) that may predict diagnostic yield and complication rate, which may make the decision to choose up-front between diagnostic modalities easier, as regards CT-GNB vs. TBB. 

## 2. Materials and Methods

### 2.1. Patient Selection

The study was conducted in accordance with the Declaration of Helsinki and was reviewed and approved by the Ethics Committee of Juntendo University Hospital, Tokyo, Japan (IRB number 18-083). The requirement for informed consent was waived owing to the retrospective design of the study. We retrospectively enrolled and reviewed 110 consecutive patients who underwent chest CT-GNB for subpleural lesions or intrapulmonary targets, defined as targets surrounded by normal lung parenchyma, at Juntendo University Hospital between February 2009 and June 2019. The medical records of all patients were reviewed for information such as age, sex, body mass index (BMI), smoking history, comorbidities of their lungs, pathological diagnostic rate, and position during CT-GNB. The size of each lung tumor was measured based on its mean diameter using the axial lung window setting. The needle length at the time of biopsy was defined in three ways: operative proximal needle length from skin to the outer border of the tumor (Length A), operative in-tumor needle length (Length B), and operative distal needle length from the outer border of tumor (Length C) (Figure S1). The presence or absence of a bronchus sign (the presence of a bronchus directly leading to the target on CT) was recorded to compare the diagnostic rate of TBB (Figure S2B). Thus, images of the target were assessed for the following four features: (1) bronchus sign (Figure S2A), (2) inhomogeneous tumor (Figure S2C), (3) GGO (Figure S2E), (4) subpleural lesion (Figure S2F). Complications occurring after CT-GNB procedures were also assessed. An inhomogeneous tumor was defined as a tumor suspected of having necrotic tissue inside and showing different levels of HU in different regions of the tumor. 

Furthermore, we retrospectively enrolled and reviewed 988 consecutive patients who were suspected of lung cancer and thus underwent bronchoscopy at Juntendo University Hospital during the same period to compare the usefulness of bronchoscopy as the first diagnostic modality. Of these, 297 patients with mediastinal or hilar lymphadenopathy who underwent synchronous endobronchial ultrasound-guided transbronchial needle aspiration (EBUS-TBNA) or endoscopic ultrasound-guided fine needle aspiration (EUS-FNA) were excluded. In addition, 144 patients who did not undergo endobronchial ultrasound-guided transbronchial biopsy with a guide sheath (EBUS-GS) were excluded. No patients underwent transbronchial tumor biopsy by TBB with a navigation system or electromagnetic navigational bronchoscopy [18]. For the remaining 547 patients who underwent TBB, the diagnostic rate, images of targets, and complications were investigated. When the results were nondiagnostic, the patient was referred for repeated TBB, CT-GNB, chest ultrasound-guided percutaneous needle biopsy (US-GNB), or thoracoscopic surgery (Figure S3). Complications that occurred during or after each procedure were recorded.

### 2.2. Biopsy Modality

CT-GNB was performed by two radiologists with > 3 years of experience. All CT scans were reviewed by a chest radiologist and pulmonologist with ≥10 years of experience in the interpretation of thoracic CT. All biopsies were performed under CT guidance using a 4-slice CT (Asterion 4, Toshiba, Tokyo, Japan) or a 16-slice CT (Aquilion 16, Toshiba, Tokyo, Japan). At the time of biopsy, selected images were obtained of the required area using 2 mm slice thickness with standard lung window and mediastinum window acquired during the procedure. Biopsies were planned to avoid bone, artery, bulla, and pleural surfaces. Procedures were performed with patients in the prone, supine, or lateral decubitus position, depending on the location of the target. After sterile preparation, local anesthesia was induced with a subcutaneous injection of 1% xylocaine. Biopsies were performed using the coaxial method with an 18-gauge or 20-gauge introducer needle (Argon Medical Devices, Inc. Tru Core Biopsy Instrument 1445 Flat Creek Road Athens, Texas 7571, USA) on integrated introducer and automated biopsy instruments. Patients were instructed to hold their breath when the needle penetrated the pleura. After confirmation on the CT images that the tip of the needle had reached the tumor, the operator removed the stylet of the introducer needle and introduced an automated biopsy instrument. The number of samples varied depending on the size of the sample and the patient’s condition. Most specimens were preserved in 10% formalin and submitted to the pathology department, but some specimens were submitted to READ specimens. Whether a READ specimen was submitted depended on the suspected hematological disease. CT was performed immediately after the procedure to detect complications. Complications such as pneumothorax and hemorrhage in the lung were recorded. All patients were required to undergo bed rest for 4 h in the ward after the biopsy. After the 4 h of bed rest, they were taken for chest radiography, and if there were no complications, their bed rest level was elevated. The next day, another chest radiograph was obtained from all patients to check for delayed-onset complications. Lung hemorrhage was detected around the tumor or inside the lung parenchyma through which the needle passed in the CT images at the end of the CT-GNB as the CT-GNB unique complication.

TBB and all related examinations were conducted by 7–11 pulmonologists with 3 years of experience in performing radial probe endobronchial ultrasound. TBB procedures were performed under local anesthesia at the pharynx by spraying 1% xylocaine and with conscious sedation with intravenous midazolam and fentanyl. All TBB procedures were performed with intrabronchial administration of 1% xylocaine. During the procedure, patients received supplemental oxygen through a nasal catheter, and vital signs were monitored. An EBUS-GS device was used with one of the following combinations of bronchoscopes and guide sheath kits (Olympus Corporation, Shinjuku-ku, Tokyo, Japan), depending on the size and location of the target: BF-p260F or BF-p290 (working channel diameter 2.0 mm) with a K201 guide sheath kit equipped with biopsy forceps (FB233D, outer diameter 1.5 mm) and a cytology brush (BC-204D-2010, outer diameter 1.4 mm). Before the procedure, we identified the relevant bronchus for the target and evaluated the CT scan bronchus sign on a thin-slice section of chest CT images. We introduced a guide sheath into the target via the relevant bronchus and inserted an EBUS probe via the guide sheath. After obtaining proper EBUS findings (within or adjacent to the target), we performed several biopsies and conducted bronchial brush cytology and bronchial washing. The number of samples varied and depended on the size of the samples and the patient’s condition. After the biopsy procedure, we confirmed that bleeding from the bronchus had stopped and that there was no pneumothorax on fluoroscopic images. Bleeding from bronchus was defined by intrabronchial hemorrhage from biopsied bronchus that was observable by bronchoscopy as the unique complication of TBB.

### 2.3. Pathological Examination

All pathological results were determined by two experienced pathologists who performed cytological (brush smear and alveolar lavage) or histological (forceps biopsy) analysis. A definite diagnosis was recorded from which to calculate the diagnostic yield. A definite diagnosis was either proof of malignancy or a defined benign pathology (e.g., tuberculosis) on histological, cytological, or bacteriological analysis. When results were nondiagnostic, such as with non-specific inflammatory changes, the patient was referred for biopsy, including repetition of biopsy modalities. Patients were followed up for at least 2 years if they refused further examination.

### 2.4. Statistical Analysis

To compare the availability and rate of complications of CT-GNB and TBB, we investigated two groups depending on which modality led to a successful diagnosis: the CT-GNB group and the TBB group. The χ^2^ test and Pearson’s chi-square test were used to compare the factors. The significance threshold of the *p*-value was set at *p* < 0.05. Statistical analysis was performed using JMP software (JMP v.10, SAS Institute, Cary, NC, USA). 

## 3. Results

### 3.1. Patient Characteristics

The characteristics of the patients are listed in Table 1. Of the 110 CT-GNB patients, 55 were men and 55 were women. The median age was 65 years, and the median BMI was 21.7. There were 56 patients who had a smoking history, including ex-smokers. The comorbid pulmonary diseases included chronic obstructive pulmonary disease (COPD) in 43 patients, interstitial pneumonia (IP) in 10 patients, nontuberculous myco-bacteriosis (NTM) in two patients, obsolete tuberculosis in three patients, and other lung diseases in four patients. There were four patients with concurrent COPD and IP, combined pulmonary fibrosis and emphysema (CPFE). A definitive diagnosis rate of CT-GNB was obtained in 96 (87.3%) patients. The pathological diagnoses included primary lung cancer in 61 (55.5%) patients, metastatic lung cancer in 17 (15.5%) patients, other malignancies involving classical Hodgkin lymphoma, bronchus-associated lymphoid tissue (BALT) lymphoma in five patients, and benign disease involving hamartoma, mycobacterium infection, and inflammatory pseudotumor in 13 patients. Among the patients with metastatic lung cancer, three had pulmonary metastasis from breast cancer, two from kidney cancer (clear cell carcinoma), and two from thyroid cancer. There was one patient each with pulmonary metastasis from sarcoma, bladder cancer, meso-pharynx cancer, cholangiocarcinoma, cervical cancer, duodenal cancer, pancreatic cancer, esophageal cancer, prostate cancer, and colon cancer. There were 14 patients classified as “Others” with no definitive diagnosis. Of these 14 patients, seven had nonspecific inflammation, five had alveolar tissue, one had necrotic tissue, and one had insufficient material. The definitive diagnosis rate of CT-GNB and TBB in only primary and metastatic lung cancer cases were 84.8% and 75.3% respectively, and there was statistically no difference between the groups. At the time of CT-guided biopsy, 32 patients were in the supine position, 77 in the prone position, and one in the left lateral position.

### 3.2. Diagnostic Flow of Biopsy Modality

**Figure 1:**
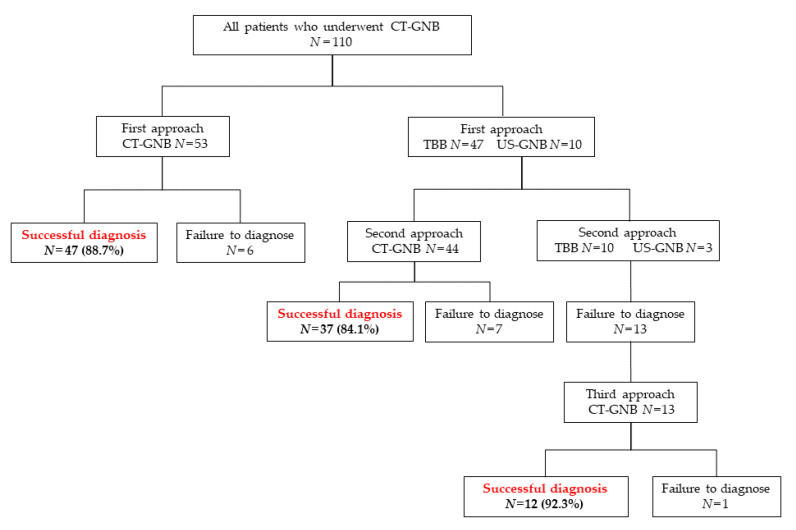
Diagnostic flow of biopsy modality. CT-GNB: Computed tomography-guided needle biopsy, TBB: Transbronchial biopsy, US-GNB: ultrasonography-guided biopsy.

A total of 110 patients underwent CT-GNB examination. Of these, 53 underwent CT-GNB for the initial diagnostic approach, and definitive diagnoses were obtained in 47 (88.7%) of these 53 patients. There were 47 and 10 patients who underwent TBB and US-GNB as the initial diagnostic approach, respectively. Furthermore, 44 of these 57 patients underwent CT-GNB for the second diagnostic approach, and 37 (84.1%) of these 44 patients achieved a high definitive diagnosis rate. Of the 57 patients who underwent TBB or US-GNB as the initial diagnostic approach, 13 patients did not choose CT-GNB for the second diagnostic approach and repeated either TBB or US-GNB. All 13 of these patients had failed diagnosis, but 12 of these 13 were successfully diagnosed by CT-GNB as the third biopsy approach. None of the patients were chosen for CT-GNB after the third diagnostic approach (Figure 1). 

### 3.3. Characteristics of the Targets

The characteristics of the targets are listed in Table 2. The targets were located in the right upper lobe in 25 patients, the right middle lobe in five patients, the right lower lobe in 29 patients, and in both the left upper and lower lobes in 25 patients. The target location was not clarified in one patient due to collapsed lung. The median long and short target diameters were 23.5 mm (7.5–111.1 mm) and 17.8 mm (6.8–72.4 mm), respectively. There were 63 (57.3%) of 110 patients who showed the presence of a bronchus inside the target in the pre-biopsy CT images, indicating a positive bronchus sign. Inhomogeneous tumors were present in 23 (20.9%) of the 110 patients. GGO alone and GGO containing partial solid targets were present in 30 (27.2%) of the 110 patients. Subpleural lesions were present in 38 (34.5%) of the 110 patients. The median operative proximal needle length from skin to the outer border of the tumor (Length A) was 77.3 mm (35.2–129.2 mm), the median operative in-tumor needle length (Length B) was 16.7 mm (5.2–47.2 mm), and the median operative distal needle length from the outer border of tumor (Length C) was 9.0 mm (0–37.1 mm).

### 3.4. Diagnostic Rate of CT-Guided Biopsies in Relation to Distance Measurements and Location of Target

We also assessed whether the diagnostic rate depended on the distance from the epidermis to the tumor (Length A) or the length punctured by the needle (Length B and C). Measured lengths were categorized into one of two groups depending on whether they were above or below a cut-off length. The cut-offs were 50 mm for Length A, 15 mm for Length B, and 10 mm for Length C. There were no significant differences between any parameters based on the cut-off lengths when assessed with χ-square tests. In all 10 (100%) of the patients where Length A was less than 50 mm, successful definitive diagnoses were obtained, although there is a small sample size for this result. Other length parameters had a definitive diagnosis rate of over 85%. We then analyzed the definitive diagnosis rate according to the different locations of the targets. The diagnostic rate was assessed based on the target location using χ-square tests and whilst there was a trend toward lower rates in the right upper and right lower lobes, none of the differences were statistically significant (Table 3). 

### 3.5. Diagnostic Rate of CT-Guided Biopsy and Bronchoscopy Based on Tumor Image Features

We classified patients based on imaging characteristics and examined the difference in diagnostic rates between CT-GNB and TBB. A total of 547 patients who underwent TBB had a definite diagnosis of lung cancer at our hospital. Patients with specific imaging characteristics were selected for review. The target CT images reviewed by a chest radiologist and pulmonologist provide the most useful information with regard to why a biopsy choice was recommended. A pulmonologist tends to avoid bronchoscopy in cases where the bronchus sign is negative. However, in some cases it was difficult to judge from CT images whether the bronchus was present or absent in the target, and therefore six patients with negative bronchus sign underwent TBB. All of these failed to be diagnosed by TBB, and the final diagnoses were made by CT-GNB. In contrast, 42 of the 47 patients who underwent CT-GNB achieved a statistically high definitive diagnosis rate of lung cancer even in cases with a negative bronchus sign (*p* < 0.0001, 89.4%). Of the 23 patients with inhomogeneous tumors who underwent CT-GNB, 20 (87.0%) received a definitive diagnosis, and there was no statistically significant difference in the definitive diagnosis rate between the TBB group and the CT-GNB group (*p* = 0.714: 84.0%). Of the 30 patients who underwent CT-GNB and showed GGO, 23 (76.7%) received a definitive diagnosis, and there was no statistically significant difference in the definitive diagnosis rate between the TBB group and the CT-GNB group (*p* = 0.401: 68.4%). Of the 38 patients who underwent CT-GNB and showed subpleural lesions, 32 (84.2%) received a definitive diagnosis, and there was no statistically significant difference in the definitive diagnosis rate between the TBB group and the CT-GNB group (*p* = 0.305, 76.8%) (Table 4). 

### 3.6. Complications of CT-Guided Biopsy Compared to Bronchoscopy

Complications encountered with CT-GNB and TBB are listed in Table 5. Pneumothorax, which required therapeutic intervention, was defined as major pneumothorax. Of the 110 patients who underwent CT-GNB, 45 (40.9%) had no complications. The most common complication of CT-GNB was hemorrhage in the lung (46.4%), followed by pneumothorax (16.3%). Both of these complications occurred in six patients each. Of the 547 patients who underwent TBB, 458 (83.7%) had no complications. The rate of patients with no complications was higher with TBB than with CT-GNB (83.7% vs. 40.9%, respectively). One of the two patients who had severe hemoptysis after CT-GNB died of acute respiratory failure. Bleeding from bronchus was the most common complication of TBB and was detected in 68 patients (12.4%), whilst pneumothorax was present in six patients (0.9%) (Table 5). 

### 3.7. Complications with CT-Guided Biopsy Depending on Target Parameters

Next, we examined whether the incidence rate of complications varied depending on the distance to the target, target location, or imaging features of the target on CT scans. Regarding the distance to target, there were no statistically significant differences in the incidence rate of complications with Length A or C. However, when Length B was 15 mm or less, there was a statistically higher risk of a complication occurring (*p* = 0.020) compared to the other distance parameters. When the length was less than 15 mm, Length B was roughly equivalent to the actual size of the target (data not shown: *p* = 0.113). The major complications in this group were hemorrhage (82.1%) and pneumothorax (28.6%). Regarding target location, there was no statistically significant difference in the incidence rate of complications. With target image features, there were no statistically significant differences in the incidence rate of complications with bronchus sign or GGO. However, there was a statistically lower risk of complications in patients with inhomogeneous tumors and subpleural lesions compared to other image features (*p* = 0.008 and *p* < 0.0001, respectively) (Table 6). Patients presenting with GGO targets tended to have a higher incidence of complications than those without GGO targets, but the difference was not statistically significant. The major complication in the GGO group was hemorrhage (50%). The two patients who presented with hemoptysis were included in this group. Moreover, of the 43 patients with COPD who underwent CT-GNB, complications occurred in 23 patients (53.5%); of the 10 IP patients who underwent CT-GNB, complications occurred in six patients (60%). There was no statistically significant difference (*p* = 0.709). 

## 4. Discussion

In this study, the diagnostic rate of CT-GNB was 87.3%, which was higher than that of TBB (75.7%). Patients who failed diagnosis by the other biopsy modalities were finally diagnosed by CT-GNB. As if to confirm our findings, the diagnostic rate with repeated TBB was lower than that with finally performed CT-GNB. These findings suggest that CT-GNB is a reliable biopsy modality for definitive pathological diagnosis when other biopsy modalities fail to provide a diagnosis. Notably, when the images of the target had a negative bronchial sign, there was a statistically higher diagnostic rate with CT-GNB compared to that with TBB. The present results support previous studies showing that intrapulmonary lesions with a negative bronchus sign are difficult to diagnose by TBB [7]. A recent study demonstrated a peripheral target approach with negative bronchus sign cases by EBUS-FNA [19,20]. However, when using needle aspiration, it is difficult to obtain a sufficient amount of tumor tissue for pathological analysis and many oncogene mutation analyses (such as cancer panels), compared to when using CT-GNB [21]. As such, CT-GNB should be selected as the first choice of biopsy modality over TBB in cases where the bronchus sign is negative. 

In the present study, the complication rate with CT-GNB was higher than that with TBB (59.1% compared to 4.0%, respectively), suggesting that careful consideration should be given before selecting CT-GNB as the diagnostic modality. But, with an inhomogeneous tumor, subpleural lesion, and when more than 15 mm of punctured needle length was within the target, there were fewer complications. The complication rate with CT-GNB in the present study was slightly higher than has been previously reported [22,23]. It may have been the case that an increased length of needle puncture (>50 mm, defined as Length A) (90.9%) compared to previous reports [22,23] led to an increased rate of hemorrhage and a lower rate of subpleural lesions (34.5%), resulting in the decreased rate of pneumothorax (50%) [1]. In addition, Length B, defined as the operative needle length inside the tumor, was roughly equivalent to the actual size of the target in the present study. Whilst a previous study reported that CT-GNB tends to be safer to perform when lesion lengths are larger than 15 mm [24], we did not observe any difference in the rate of complications based on size of the target. This could be important information to aid the operator in judging how far to puncture the target with CT-GNB. Furthermore, our findings are in agreement with those of previous reports that have shown fewer complications when CT-GNB is used for subpleural lesions, where there is a shorter distance between the pleura and the target [25,26]. Inhomogeneous tumors on pre-biopsy CT images, which are often associated with internal necrosis of the tumor, are associated with a lower risk of complications compared to when a necrosis pattern is not present [27]. Previous studies had only reported that there was no difference in the risk of complications, including hemoptysis, when CT-GNB was used for patients with cavities or necrosis within the lesion [28,29].

This study has several limitations. This was a single-center, retrospective study with a small number of patients. The patients who were included in the TBB/CT-GNB comparison were patients with a diagnosis of primary lung cancer or metastatic lung cancer, but the patients with a definitive diagnosis by CT-GNB included benign and other diseases. Therefore, the exclusion of patients with benign and other diseases may introduce some selection bias. Since there are no strict indication criteria for choosing between CT-GNB and TBB, and no clinical guidelines have been established, the selection bias in choosing the biopsy modality and its device, including EBUS-GS, depended on the decision of the attending doctor according to the appearance of the targets on pre-biopsy CT images. 

## 5. Conclusions

CT-GNB is an effective and reliable biopsy modality with a high diagnostic rate but also a high complication rate. CT-GNB should particularly be recommended when the bronchus sign is negative and can be safely applied in cases where the needle puncture length and pre-biopsy CT image features, such as inhomogeneous tumors and subpleural lesions, indicate a lower risk of complications.

## Figures and Tables

**Table 1:** Patient characteristics.

Median Age (Years)	65 [13–89]	
Median BMI	21.7 [14.2–35.2]	
	Number of patients	%
Sex		
Male	55	50.0%
Female	55	50.0%
Smoking history		
(+)	56	51.0%
(−)	54	49.0%
Comorbidities		
All	55	50.0%
COPD	43	39.1%
IP	10	9.1%
NTM	2	1.8%
Old TB	3	2.7%
Others	4	3.6%
Diagnosis by CT-GNB		
(+)	96	87.3%
(−)	14	12.7%
Diagnosis		
Primary lung cancer	61	55.5%
Metastatic lung cancer	17	15.5%
Other malignancies	5	4.5%
Benign disease	13	11.8%
Others	14	12.7%
Patient position		
Supine	32	29.1%
Prone	77	70.0%
Left lateral	1	0.9%

BMI: Body mass index, COPD: Chronic obstructive pulmonary disease, IP: Interstitial pneumonia, NTM: Nontuberculous myco-bacteriosis, Tb: Tuberculosis, CT-GNB: Computed tomography-guided needle biopsy.

**Table 2:** Characteristics of parenchymal lung target parameters.

	Number of Patients	%
Location of lesion		
Right upper lobe	25	22.7%
Right middle lobe	5	4.6%
Right lower lobe	29	26.3%
Left upper lobe	25	22.7%
Left lower lobe	25	22.7%
Unknown	1	0.9%
Diameter of tumor (mm)		
Median long diameter	23.5 [7.5–111.1]	
Median short diameter	17.8 [6.8–72.4]	
Characteristics of target		
Bronchus sign		
(+)	63	57.3%
(−)	47	42.7%
Inhomogeneous tumor		
(+)	23	20.9%
(−)	87	79.1%
GGO lesion		
(+)	30	27.2%
(−)	80	72.8%
Subpleural lesion		
(+)	38	34.5%
(−)	72	65.5%
Length A (mm)		
Median length	77.3 [35.2–129.2]	
Length B (mm)		
Median length	16.7 [5.2–47.2]	
Length C (mm)		
Median length	9.0 [0–37.1]	

Length A: Operative proximal needle length from skin to the outer border of the tumor, Length B: Operative in-tumor needle length, Length C: Operative distal needle length from the outer border of the tumor.

**Table 3:** Diagnostic rate by CT-GNB in relation to distance measurements and location of target.

	Number of Patients	Diagnostic Rate		* p *
		Number of patients	%	
All	110	96	87.3%	
Length A				0.205
>50 mm	100	86	86.0%	
≤50 mm	10	10	100%	
Length B				0.957
>15 mm	70	61	87.1%	
≤15 mm	40	35	87.5%	
Length C				0.607
>10 mm	48	41	85.4%	
≤10 mm	62	55	88.7%	
Location of lesion				
Right upper lobe	25	20	80.0%	0.215
Right middle lobe	5	5	100%	0.382
Right lower lobe	29	23	79.3%	0.134
Left upper lobe	25	24	96.0%	0.136
Left lower lobe	25	23	92.0%	0.420
Unknown	1	1	100%	0.701

**Table 4:** Diagnostic rate of CT-GNB and TBB in relation to various image characteristics.

	CT-GNB			TBB			*p*
	Diagnostic Rate						
Image characteristics	Number of patients	Success to diagnose	%	Number of patients	Success to diagnose	%	
Negative bronchus sign	47	42	89.4%	6	0	0%	<0.001
Inhomogeneous tumor	23	20	87.0%	194	163	84.0%	0.714
GGO lesion	30	23	76.7%	76	52	68.4%	0.401
Subpleural lesion	38	32	84.2%	263	202	76.8%	0.305

**Table 5:** Occurrence of complications with CT-GNB and TBB.

Details of Complication	CT-GNB (*n* = 110)	%	TBB (*n* = 547)	%
All	65	59.1%	89	16.3%
Pneumothorax	16	14.5%	5	0.9%
Major pneumothorax	2	1.8%	1	0.2%
Bleeding from bronchus	-	-	68	12.4%
Hemorrhage in the lung	51	46.4%	-	-
Hemoptysis	2	1.8%	2	0.4%
Death	1	0.9%	0	0%
Others	0	0.0%	14	2.6%

Major pneumothorax: pneumothorax that required therapeutic intervention.

**Table 6:** Complications of CT-GNB in relation to target parameters.

All	110			
	Complication (+)			
	Number of patients		%	*p*
Length A				0.951
>50 mm	100	59	59.0%	
≤50 mm	10	6	60.0%	
Length B				0.020
>15 mm	70	33	47.1%	
≤15 mm	40	28	70.0%	
Length C				0.522
>10 mm	48	30	62.5%	
≤10 mm	62	35	56.5%	
Location of lesion				
Right upper lobe	25	16	64.0%	0.570
Right middle lobe	5	2	40.0%	0.374
Right lower lobe	29	16	55.1%	0.617
Left upper lobe	25	17	68.0%	0.303
Left lower lobe	25	14	56.0%	0.721
Unknown	1	1	100%	0.403
Image characteristics				
Bronchus sign				0.487
(+)	63	39	61.9%	
(−)	47	26	55.3%	
Inhomogeneous tumor				
(+)	23	8	34.8%	0.008
(−)	87	57	65.5%	
GGO lesion				0.154
(+)	30	21	70.0%	
(−)	80	44	55.0%	
Subpleural lesion				<0.001
(+)	38	13	34.2%	
(−)	72	52	72.2%	

## Supplementary Materials

The following are available online at https://www.mdpi.com/article/10.3390/jcm10092031/s1, Figure S1: Definition of various lengths at time of biopsy sampling, Figure S2: Concrete example of features of lesions, Figure S3: Diagnostic flow of biopsy modality for patients who underwent TBB.
