# Supplementary figures and images for: CT Guided Needle Biopsy of Peripheral Lesions–Lesion Characteristics That May Increase the Diagnostic Yield and Reduce the Complication Rate
Source: J Clin Med. 2021 May 9;10(9):2031. doi: 10.3390/jcm10092031 (PMC8126034; doi:10.3390/jcm10092031)

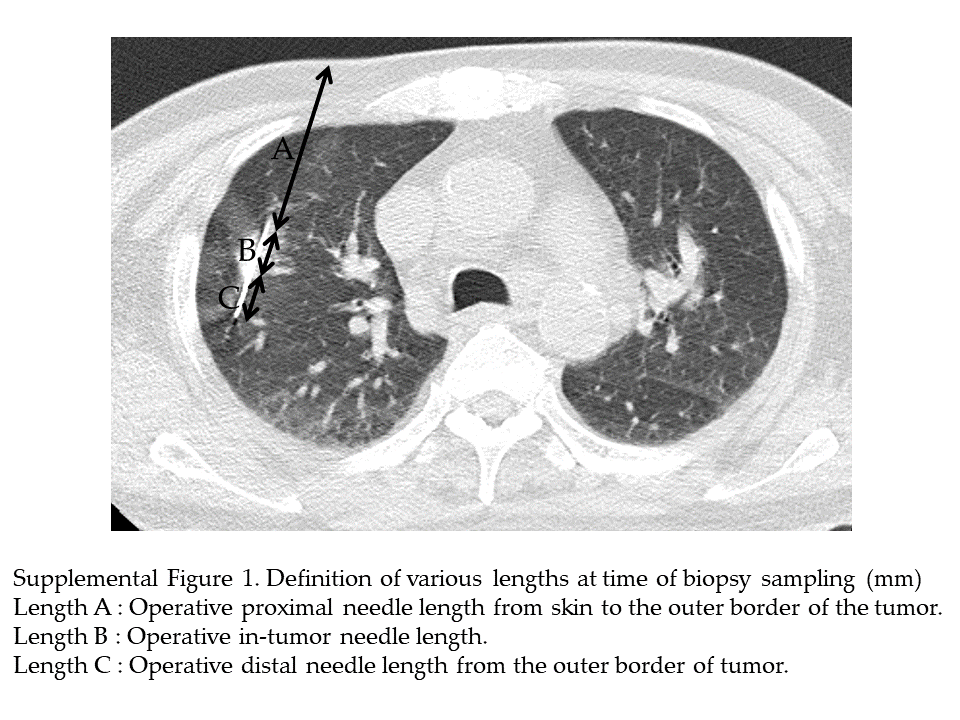

Supplement: Supplementary file 1 [file jcm-10-02031-s001.zip › jcm-1135373-supplementary 20210508/Supplemental figure1.PNG]

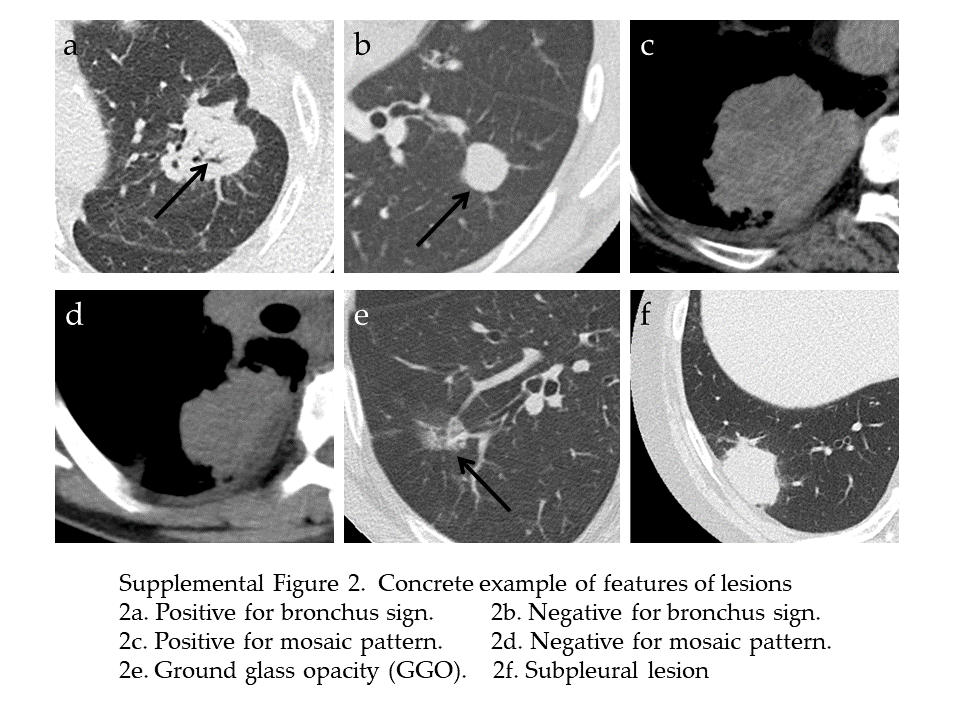

Supplement: Supplementary file 1 [file jcm-10-02031-s001.zip › jcm-1135373-supplementary 20210508/Supplemental figure2.PNG]

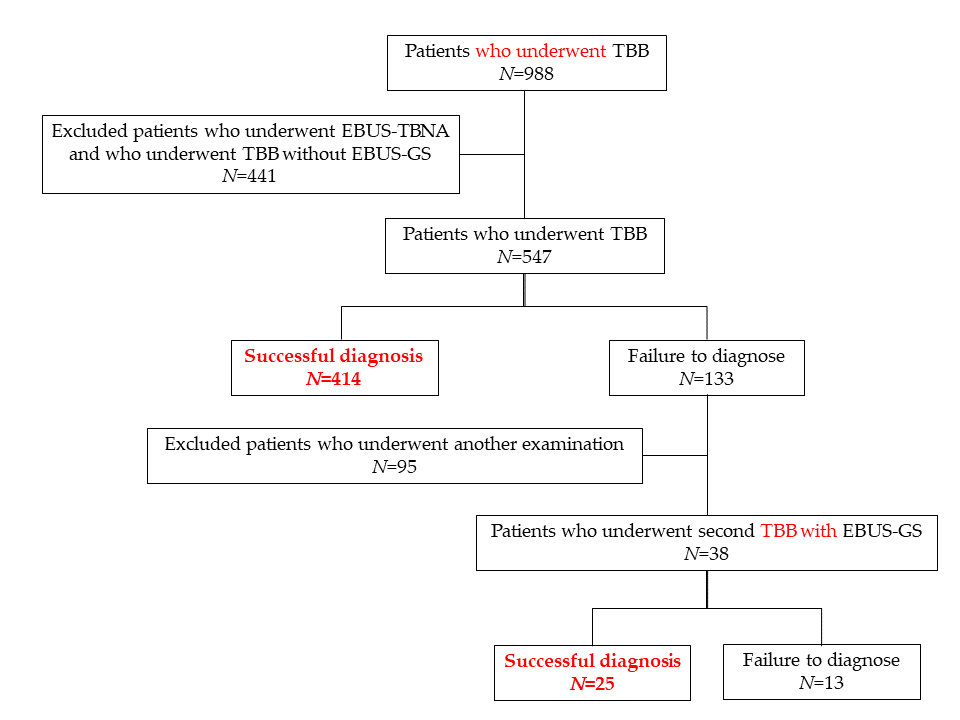

Supplement: Supplementary file 1 [file jcm-10-02031-s001.zip › jcm-1135373-supplementary 20210508/Supplemental figure3.PNG]
